# Pulmonary Hemorrhage Secondary to Superior Vena Cava Thrombosis Mimicking Upper Gastrointestinal Bleeding: A Case Report

**DOI:** 10.7759/cureus.108063

**Published:** 2026-04-30

**Authors:** Oscar Molina Lizama, Andrea Maryant Escobar, Carlos E Núñez Vásquez, Maria Ramirez, Ana Cristina Barranco Monterrosa, Jonathan J Calderón

**Affiliations:** 1 General Medicine, University of El Salvador, San Miguel, SLV; 2 Medicine, Facultad de Ciencias Médicas "Coronel y Dr. Juan Ignacio Gutiérrez Sacasa", Managua, NIC; 3 Medicine, National Autonomous University of Honduras, Tegucigalpa, HND; 4 Medicine, Universidad del Sinú, Cartagena, COL; 5 Medicine, Universidad Libre, Facultad de Ciencias de la Salud, Barranquilla, COL; 6 General Medicine, Pontificia Universidad Católica del Ecuador, Quito, ECU

**Keywords:** chronic kidney disease, hematemesis, hemodialysis, hemoptysis, superior vena cava thrombosis, upper gastrointestinal bleeding

## Abstract

Upper gastrointestinal bleeding (UGIB) is a frequent cause of emergency department admission and is most commonly attributed to peptic ulcer disease or portal hypertension-related varices. In critically ill patients, however, pulmonary bleeding may be misinterpreted as hematemesis when blood is swallowed or expelled during retching or airway compromise. In patients undergoing long-term hemodialysis, catheter-related central venous thrombosis can precipitate atypical bleeding presentations. We report the case of a 45-year-old man with chronic kidney disease on maintenance hemodialysis who presented with sudden-onset recurrent hematemesis and hemodynamic instability. Initial evaluation suggested severe UGIB, prompting endotracheal intubation, vasopressor support, and urgent upper endoscopy. Endoscopy demonstrated small esophageal varices and erosive gastropathy without evidence of active bleeding. Subsequent chest computed tomography revealed superior vena cava thrombosis with extensive mediastinal collateral circulation and imaging findings consistent with pulmonary hemorrhage. Bronchoscopy confirmed active bleeding originating from the right bronchial tree, establishing the diagnosis of pulmonary hemorrhage secondary to superior vena cava thrombosis. This case underscores an important diagnostic pitfall: severe pulmonary hemorrhage may mimic UGIB, particularly when initial endoscopic evaluation is non-diagnostic. In hemodialysis patients, superior vena cava thrombosis and collateral vessel formation should be considered as potential mechanisms of airway bleeding even in the absence of classic superior vena cava syndrome. Early thoracic imaging and bronchoscopy are essential when endoscopic findings do not account for persistent or severe hematemesis to prevent diagnostic delays and inappropriate gastrointestinal interventions.

## Introduction

Upper gastrointestinal bleeding (UGIB) is one of the most frequent causes of admission to emergency departments. Its incidence is estimated at 40 to 150 cases per 100,000 inhabitants per year, representing a significant public health concern due to its associated morbidity, mortality, and healthcare resource utilization [1]. The most common etiologies include peptic ulcer disease, esophageal varices, and erosive lesions, and early identification is essential given the risk of hemodynamic instability and death.

However, various extraintestinal processes can mimic UGIB. Among them, massive hemoptysis and other non-gastrointestinal sources of bleeding may present clinically as hematemesis, complicating the initial diagnosis. Therefore, bleeding originating from the respiratory tract or central venous system must be considered within a broad differential diagnosis.

In patients with chronic kidney disease (CKD) undergoing hemodialysis, thrombosis associated with central venous catheters is a well-documented complication, with an incidence reported to be as high as 23%-38% in patients with multiple vascular accesses [2]. Nevertheless, involvement of the superior vena cava (SVC) and its presentation as apparent UGIB is extraordinarily uncommon. This case illustrates how SVC obstruction can lead to pulmonary hemorrhage mimicking hematemesis, underscoring the importance of considering non-gastrointestinal etiologies when initial endoscopic evaluation does not explain the clinical presentation [3].

## Case presentation

A 45-year-old male was brought to the Emergency Department (ED) with a sudden onset of hematemesis, reporting at least four episodes within the previous 30 minutes. His medical history was significant for chronic hypertension and chronic kidney disease, and he was enrolled in a hemodialysis program, receiving treatment twice weekly through a right upper extremity arteriovenous fistula. He had no history of anticoagulant use, smoking, or prior thrombotic events, and no other relevant medical history was identified. Review of systems was negative for fever, chest pain, cough, hemoptysis, or other respiratory symptoms. During his stay in the ED, the patient developed retching followed by a moderate amount of bloody emesis, which initially supported a diagnosis of upper gastrointestinal bleeding and prompted targeted management.

**Figure 1:**
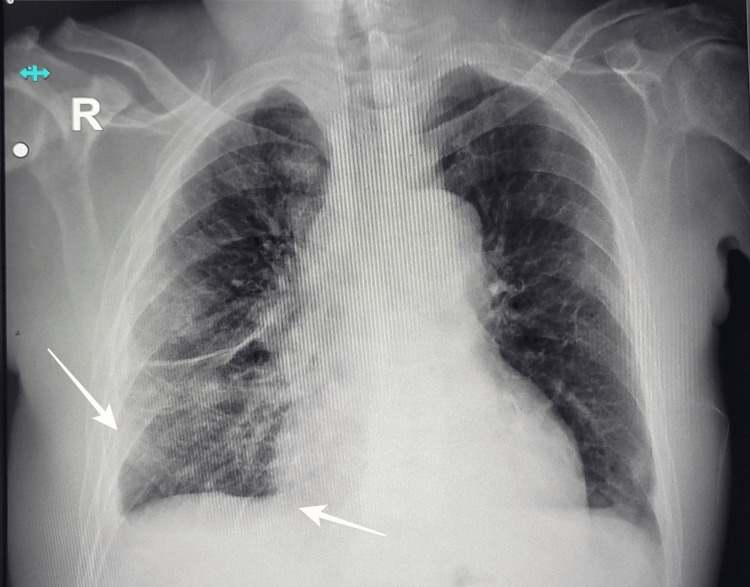
Chest radiograph demonstrating prominent bronchovascular markings (arrows), no other relevant cardiovascular or lung findings.

On admission, vital signs revealed a blood pressure of 80/60 mmHg, heart rate of 76 beats per minute, oxygen saturation of 90% on room air, and a temperature of 36.5°C. Physical examination did not reveal significant abnormalities in the cardiovascular, respiratory, or abdominal systems. The patient appeared lethargic, with minimal verbal response and poor responsiveness to stimuli. Due to his deteriorating clinical status, airway protection was achieved via endotracheal intubation, and mechanical ventilation was initiated along with intravenous vasopressor support. A chest radiograph demonstrated prominent bronchovascular markings, interlobar fissure thickening in the right middle lobe, and blunting of the right costophrenic angle suggestive of right-sided pleural effusion (Figure 1).

**Figure 2:**
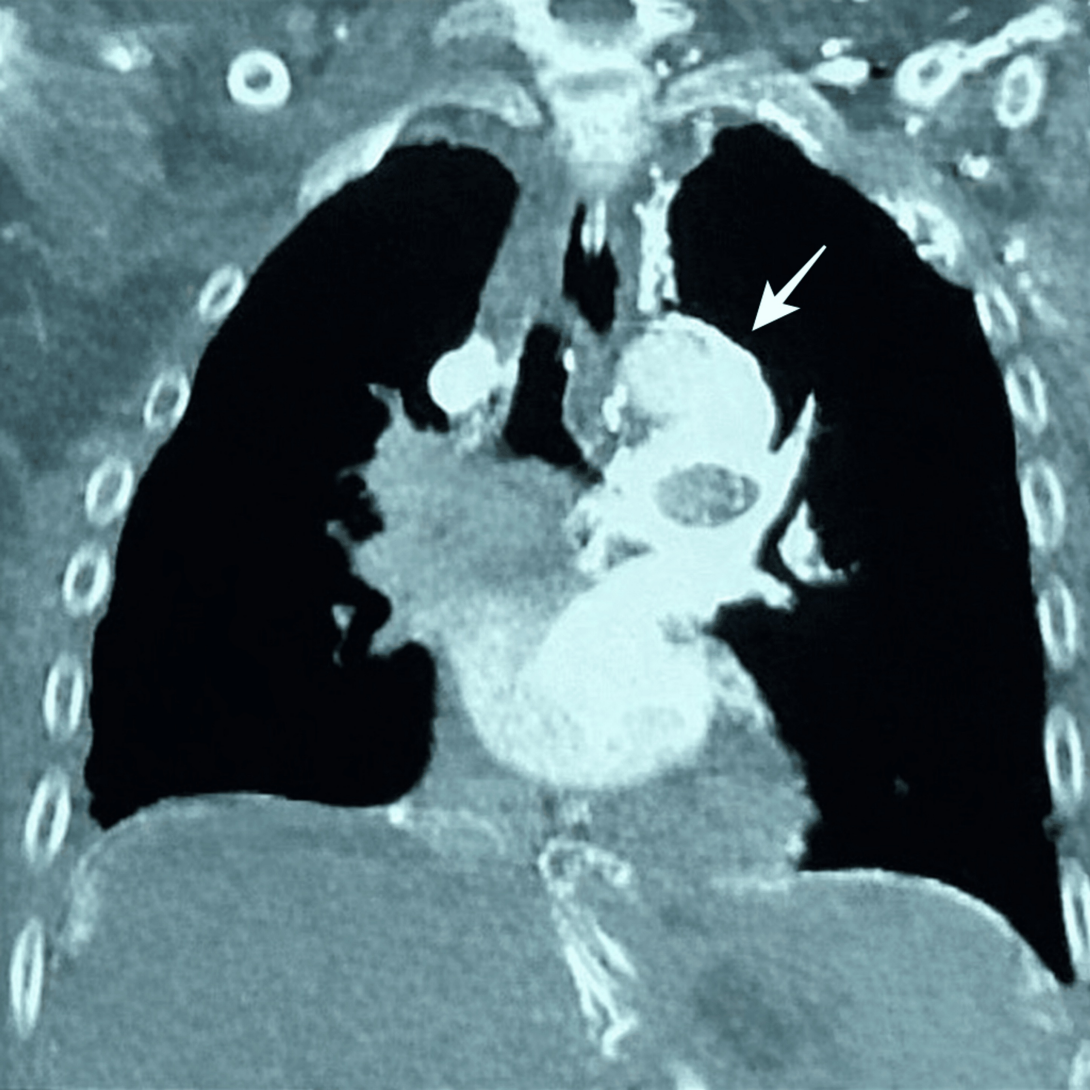
Contrast-enhanced chest computed tomography demonstrating thrombosis of the superior vena cava with extensive mediastinal collateral vessels (arrow).

The patient underwent upper endoscopy, which revealed small esophageal varices and acute erosive pangastropathy; however, no active source of gastrointestinal bleeding was identified. A chest computed tomography (CT) scan was obtained for further evaluation. Imaging demonstrated thrombosis of the superior vena cava, associated with the development of multiple mediastinal collateral vessels arising from the innominate vein, more prominent in the left perihilar region (Figure 2). The patient was not receiving anticoagulant therapy prior to presentation. During hospitalization, anticoagulation for the underlying superior vena cava thrombosis was deferred by the Critical Care team due to active bleeding and high hemorrhagic risk, pending further evaluation by Hematology.

**Figure 3:**
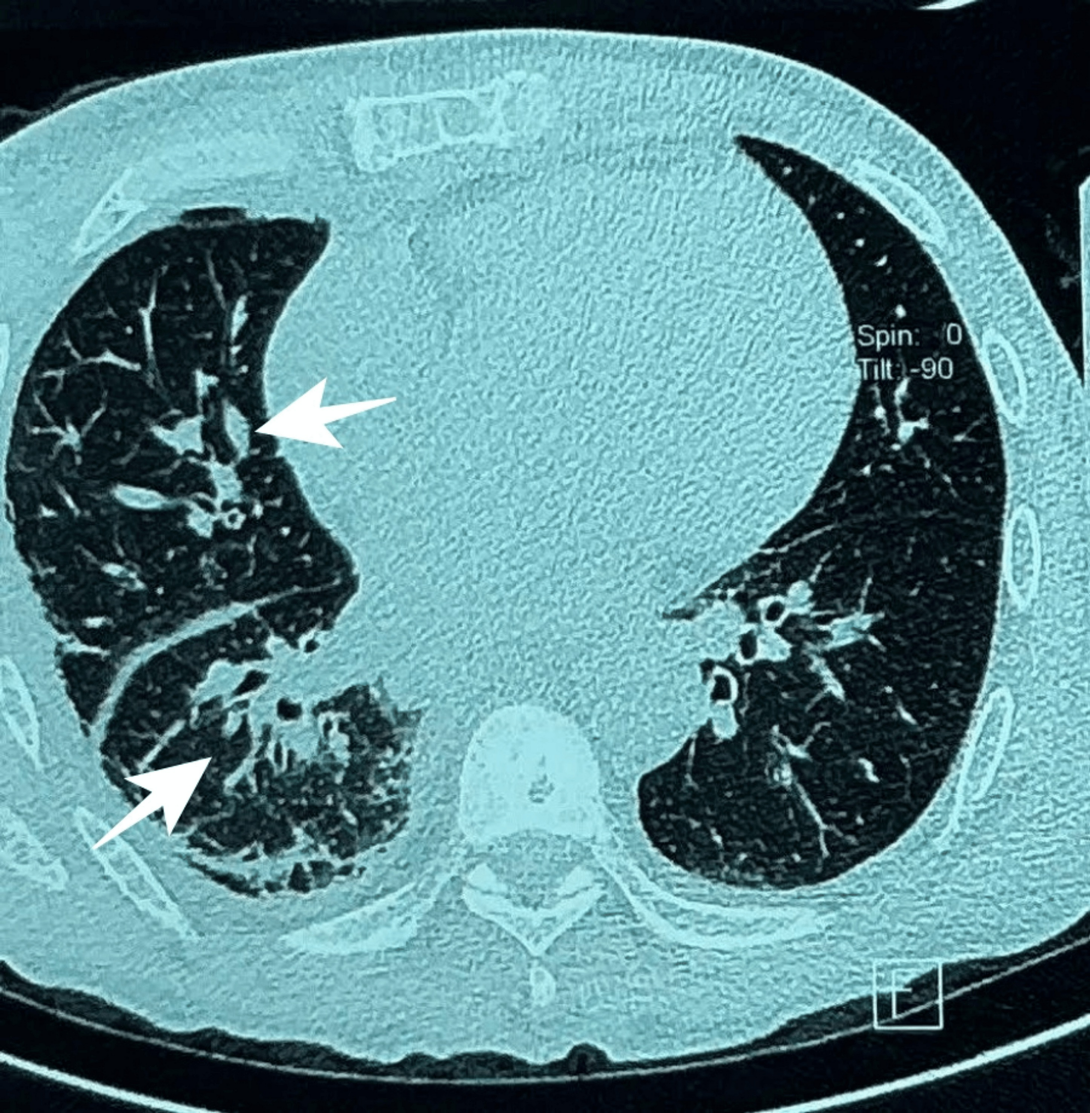
Contrast-enhanced chest CT showing areas of alveolar consolidation in the right upper lobe and lingula consistent with pulmonary hemorrhage (arrows).

**Figure 4:**
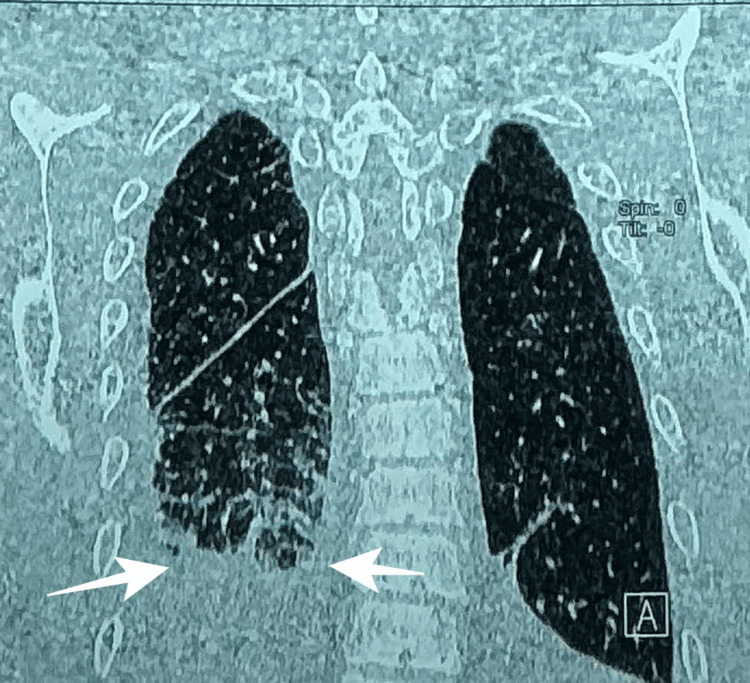
Contrast-enhanced chest CT demonstrating right-sided pleural effusion (arrows).

Additionally, multiple areas of alveolar consolidation were observed in the anterior segment of the right upper lobe and lingula, without history of pneumonia or fluid aspiration, most consistent with pulmonary hemorrhage (Figure 3). A right-sided pleural effusion was also identified (Figure 4).

**Figure 5:**
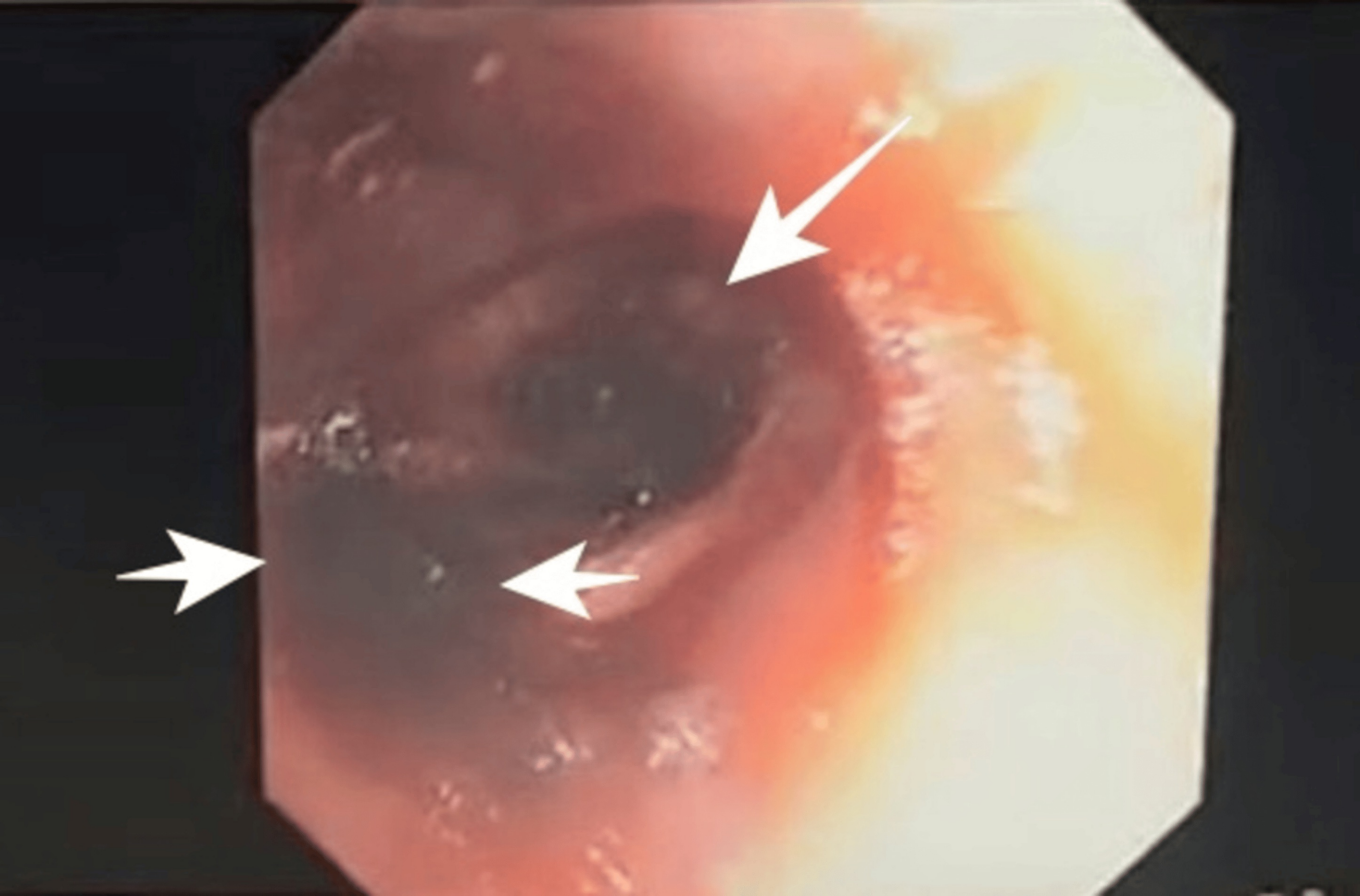
Flexible bronchoscopy demonstrating active bleeding with firmly adherent blood clots within the right bronchial tree (white arrows), confirming a pulmonary source of hemorrhage.

Due to these findings, flexible bronchoscopy was performed and showed the right bronchial tree with bloody secretions and firmly adherent clots in the bronchus, with active bleeding noted upon removal. The left bronchial tree showed scant bloody secretions without clots (Figure 5).

Laboratory findings over the first three hospital days demonstrated progressive anemia, thrombocytopenia, worsening renal function, electrolyte imbalance, and elevated inflammatory markers (Table 1).

**Table 1:** Laboratory findings. BUN: blood urea nitrogen, eGFR: estimated glomerular filtration rate, AST: aspartate aminotransferase, ALT: alanine aminotransferase, CPK: creatine phosphokinase, INR: international normalized ratio.

Parameter	Day 1	Day 2	Day 3	Normal Reference Range
Hemoglobin (g/dL)	10.4	8.6	9.4	12-17
Hematocrit (%)	32.2	27.3	29.8	37-51
White blood cell count (×10³/µL)	5.7	5.8	4.9	5.0-10.0
Neutrophils %	75	87.7	83.1	37-70
Lymphocytes %	11.4	4.2	6.7	20-50
Red blood cell count (× 10³/µL)	3.86	3.21	3.45	4.1-5.7
Platelets (×10³/µL)	177	130	109	150-400
Creatinine (mg/dL)	5.66	6.30	-	0.7-1.2
Urea, mg/dL	86.9	97.4	-	0-50
BUN, mg/dL	40.6	45.5	-	6-20
eGFR, mL/min/1.73 m²	11.13	9.78	-	90-120
Sodium (Na⁺), mEq/L	136.6	129.4	-	136-145
Potassium (K⁺), mEq/L	4.1	4.1	-	3.5-5.1
Chloride (Cl⁻), mEq/L	98	95.20	-	98-107
Calcium (Ca²⁺), mg/dL	9.4	7.9	-	8.6-10.2
Phosphorus (P), mg/dL	3.4	3.9	-	2.5-4.5
Magnesium (Mg²⁺), mg/dL	2.5	2.2	-	1.6-2.6
Glucose, mg/dL	114	266	-	74-106
Albumin, g/dL	3.4	2.8	-	3.5-5.2
AST, U/L	29.2	30.3	-	0.0-42
ALT, U/L	24.8	19.4	-	10-40
CPK, U/L	165	147	-	39-190
C-reactive protein (mg/dL)	8.0	9.1	-	00.0-0.5
Thrombin time	11.2	11.1	-	11-13.5 s
Prothrombin time	27	27.3	-	25-35 s
INR	0.92	0.93	-	0.8-1.2 s
Dimer D	540	600	-	0-230

The patient received multidisciplinary management involving multiple specialties at the hospital, including Pulmonology, Critical Care and Hematology. The team elected to defer initiation of anticoagulant therapy for 24 hours and was prepared for SVC thrombectomy. The patient showed clinical improvement and was subsequently discharged from the Intensive Care Unit.

## Discussion

This case illustrates a rare and diagnostically challenging presentation of pulmonary hemorrhage due to SVC thrombosis masquerading as hematemesis. The patient, a 45-year-old man with advanced CKD on hemodialysis, initially appeared to have a severe UGIB, supported by repeated episodes of apparent bloody emesis and hemodynamic instability. However, urgent endoscopy revealed no active gastrointestinal source, prompting reconsideration of the initial diagnosis and evaluation of extraintestinal etiologies.

This scenario illustrates a critical diagnostic pitfall in the evaluation of acute bleeding. In critically ill patients, pulmonary hemorrhage may be misinterpreted as a gastrointestinal source, particularly when blood is swallowed or expectorated during retching, intubation, or airway compromise. Recognizing this distinction is essential, as failure to do so may delay appropriate diagnostic evaluation and result in unnecessary or repetitive gastrointestinal interventions, while delaying identification of the true bleeding source and definitive management.

SVC syndrome is defined by obstruction of the SVC with subsequent venous congestion. Although malignancy has been the predominant cause, benign etiologies currently represent up to 40%. Patients with CKD on long-term hemodialysis constitute a vulnerable population owing to recurrent central venous access [4]. In this case, the lack of typical signs contributed to the initial misinterpretation of the bleeding source.

Imaging played a central role in the diagnostic approach and clinical decision-making for this patient, whose initial presentation resembled a severe UGIB. Although the patient had repeated episodes of hematemesis, early endoscopy showed only small esophageal varices and erosive pangastropathy without active bleeding. This finding is consistent with reports indicating that up to 10%-15% of UGIB cases have no identifiable source on the initial endoscopy, highlighting the need to consider alternative origins when endoscopic results are inconclusive [5].

Computed tomography (CT) significantly redirected the diagnostic evaluation by revealing SVC thrombosis, mediastinal collateral circulation, and imaging features suggestive of pulmonary hemorrhage. Fibrobronchoscopy further supported a respiratory source of bleeding by demonstrating blood-stained secretions and clots in the right bronchial tree. The combined use of CT and fibrobronchoscopy has been shown to improve localization of the bleeding source and to help distinguish hemoptysis from hematemesis, particularly in complex or critically ill patients [6,7]. From a management standpoint, imaging findings justified escalation of care, including airway protection, mechanical ventilation, vasopressor support, and targeted evaluation of both gastrointestinal and pulmonary systems. When CT suggests pulmonary hemorrhage or vascular obstruction, management should shift toward a multidisciplinary approach involving critical care, pulmonology, and interventional radiology. Imaging not only assists in diagnosis but also guides individualized therapeutic strategies, helping avoid unnecessary procedures such as repeat endoscopy when a non-gastrointestinal source is evident [8].

The mechanisms linking SVC thrombosis to pulmonary hemorrhage are not fully established, but several pathways have been proposed. One hypothesis is that venous occlusion increases proximal venous pressure, leading to dilation of mediastinal and bronchial collateral veins [9]. Congestion within these fragile vessels may predispose them to rupture under minor stressors. In our patient, the extensive mediastinal collaterals seen on CT support this mechanism. A second consideration is the baseline vascular fragility associated with CKD and its prothrombotic, endothelial dysfunction state, which increases bleeding susceptibility [10]. Additionally, repeated central venous access in hemodialysis patients contributes to venous injury, stenosis, and collateral circulation, factors that together provide a possible explanation for the hemorrhage observed in our case [11]. Most published reports describe classical features of SVC syndrome including facial swelling, upper-extremity edema, dyspnea, or prominent superficial veins, whereas hemoptysis is far less frequently reported [12]. Our case is notable because the patient lacked these typical signs, making the diagnosis initially misleading and more suggestive of an upper gastrointestinal source. This atypical presentation illustrates how pulmonary bleeding secondary to SVC thrombosis may mimic hematemesis and delay appropriate evaluation if thoracic imaging is not promptly obtained.

This observation carries important clinical implications. In hemodialysis patients with recurrent central venous access, SVC thrombosis should be considered when bleeding is unexplained or inconsistent with endoscopic findings. Early chest imaging is essential to differentiate pulmonary from gastrointestinal sources and to identify collateral circulation that may predispose to hemorrhage. Recognizing this association can guide more targeted management and prevent unnecessary or repetitive gastrointestinal procedures when a thoracic origin is more likely.

## Conclusions

This case highlights the importance of recognizing SVC thrombosis as an uncommon but clinically relevant cause of pulmonary hemorrhage that may closely mimic UGIB, particularly in patients undergoing long-term hemodialysis. When apparent hematemesis is accompanied by hemodynamic instability and endoscopic evaluation fails to identify an active gastrointestinal source, clinicians should maintain a high index of suspicion for extraintestinal etiologies, including a thoracic origin. Early consideration of thoracic imaging and bronchoscopy is essential to accurately distinguish hemoptysis from hematemesis, allowing timely diagnosis, appropriate management, and avoidance of unnecessary or repetitive gastrointestinal interventions in this vulnerable population.

## References

[1] Stanley AJ, Laine L (2019). Management of acute upper gastrointestinal bleeding. BMJ.

[2] Chemla ES, Morsy M, Anderson L, Whitemore A (2007). Inflow reduction by distalization of anastomosis treats efficiently high-inflow high-cardiac output vascular access for hemodialysis. Semin Dial.

[3] Wilson LD, Detterbeck FC, Yahalom J (2007). Superior vena cava syndrome with malignant causes. N Engl J Med.

[4] Seligson MT, Surowiec SM (2026). Superior vena cava syndrome. StatPearls [Internet].

[5] Bruzzi JF, Rémy-Jardin M, Delhaye D, Teisseire A, Khalil C, Rémy J (2006). Multi-detector row CT of hemoptysis. Radiographics.

[6] Laine L, Jensen DM (2012). Management of patients with ulcer bleeding. Am J Gastroenterol.

[7] Sakr L, Dutau H (2010). Massive hemoptysis: an update on the role of bronchoscopy in diagnosis and management. Respiration.

[8] Radchenko C, Alraiyes AH, Shojaee S (2017). A systematic approach to the management of massive hemoptysis. J Thorac Dis.

[9] Kapur S, Paik E, Rezaei A, Vu DN (2010). Where there is blood, there is a way: unusual collateral vessels in superior and inferior vena cava obstruction. Radiographics.

[10] Saeed Z, Sirolli V, Bonomini M, Gallina S, Renda G (2024). Hallmarks for thrombotic and hemorrhagic risks in chronic kidney disease patients. Int J Mol Sci.

[11] Usman S, Cheema MA, Mustafa S, Iftikhar A (2024). Fatal hemoptysis with pleural effusions secondary to superior vena cava obstruction as a complication of central venous catheterization. Cureus.

[12] Rice TW, Rodriguez RM, Light RW (2006). The superior vena cava syndrome: clinical characteristics and evolving etiology. Medicine.

